# A Survey on Deep-Learning-Based Diabetic Retinopathy Classification

**DOI:** 10.3390/diagnostics13030345

**Published:** 2023-01-18

**Authors:** Anila Sebastian, Omar Elharrouss, Somaya Al-Maadeed, Noor Almaadeed

**Affiliations:** Department of Computer Science and Engineering, Qatar University, Doha P.O. Box 2713, Qatar

**Keywords:** diabetic retinopathy grading, diabetic retinopathy detection, deep learning, convolutional neural network, retinal fundus images

## Abstract

The number of people who suffer from diabetes in the world has been considerably increasing recently. It affects people of all ages. People who have had diabetes for a long time are affected by a condition called Diabetic Retinopathy (DR), which damages the eyes. Automatic detection using new technologies for early detection can help avoid complications such as the loss of vision. Currently, with the development of Artificial Intelligence (AI) techniques, especially Deep Learning (DL), DL-based methods are widely preferred for developing DR detection systems. For this purpose, this study surveyed the existing literature on diabetic retinopathy diagnoses from fundus images using deep learning and provides a brief description of the current DL techniques that are used by researchers in this field. After that, this study lists some of the commonly used datasets. This is followed by a performance comparison of these reviewed methods with respect to some commonly used metrics in computer vision tasks.

## 1. Introduction

During the past two decades, the number of people affected by diabetes has increased alarmingly. According to the IDF Diabetes Atlas [1], almost half a billion people of all ages have been diagnosed with it across the globe. This is expected to reach seven-hundred million by 2045. It is a global health concern. The IDF Diabetes Atlas also warns that, by 2040, one in three diabetes patients will develop Diabetic Retinopathy (DR). DR is a condition that can be identified by the presence of injured blood vessels behind the retina. This may result in serious complications such as the loss of vision when it goes undetected for a long time, hence the importance of addressing this issue. At present, doctors manually examine the fundus images of the eye to assess the severity of DR. This consumes much time, and there is a shortage of available medical professionals with respect to the actual number of patients. Due to these reasons, many patients do not receive medical care in a timely manner. Even though patients suffering from diabetes are advised by physicians to receive regular medical screenings of their fundus, many cases are left undetected until the disease becomes severe [2]. Hence, it is desirable to have an automated system to help in the detection of diabetic retinopathy.

**Figure 1:**
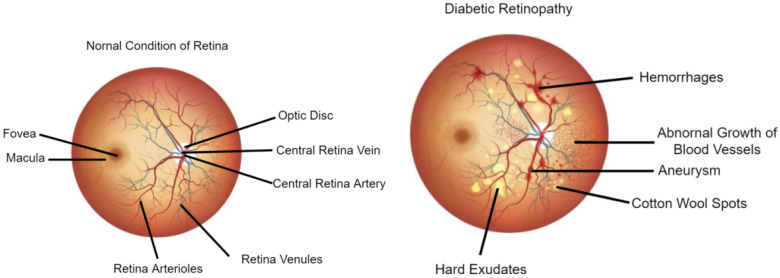
Visualization of a healthy retina and an unhealthy retina (https://neoretina.com/blog/diabetic-retinopathy-can-it-be-reversed/, accessed on 1 August 2022).

Most studies in this field use fundus images, which provide visual records that document the present ophthalmic appearance of a person’s retina. The presence of DR symptoms in these fundus images can be used to classify it using several steps such as retinal blood vessel segmentation, lesion segmentation, and DR detection [3]. The detection of DR and its current stage can be determined by investigating the presence/absence of several lesions. Some of the lesions are microaneurysms (MAs), superficial retinal hemorrhages (SRHs), exudates (Exs)—both soft exudates (SEs) and hard exudates (HEs)—intraretinal hemorrhages (IHEs), and cotton wool spots (CWSs). Figure 1 shows a comparison between a healthy retina and an unhealthy retina.

With the development of AI techniques, including machine learning and deep learning, high-performance detection and grading of the retina to detect and segment the infected parts of the retina become possible. Machine learning approaches are widely used for DR classification and grading. Nazir et al. [4] used a new way to represent fundus images called the “tetragonal local octa pattern (T-LOP) features”. Later, this classification was performed using extreme learning machine. Three ML classifiers—support vector machine (SVM), random forest, and J48—were used by the authors in [5]. The Gabor wavelet method followed by the AdaBoost classifier were used by the authors in [6] to grade DR. Recently, many deep learning techniques have been utilized by researchers to perform these tasks. This study provides a review of the present literature in this area with a focus on how DL is being used for DR detection and grading from fundus images. DL is a branch of AI that makes use of artificial neural networks with multiple processing layers to gradually extract the high-level features from the data. In this paper, we also summarize the DL architectures that have been used by the different reviewed studies.

However, significant research in this field using DL is also being carried out using optical coherence tomography (OCT) images, which have a higher resolution [7,8,9]. OCT images are more suitable than fundus images for developing systems that require micrometer resolution and a penetration depth of millimeters, which is why they are used by researchers for DR diagnosis, especially at the early stages [7].

The paper is organized as follows. The related works on DR detection and DR grading are presented in Section 2. Section 3 describes some of the preprocessing techniques that are used. Section 4 describes the datasets used. A comparison and discussion of the experiments are provided in Section 5. Some of future directions are provided in Section 6 The conclusion is presented in Section 7.

## 2. Literature Review

**Figure 2:**
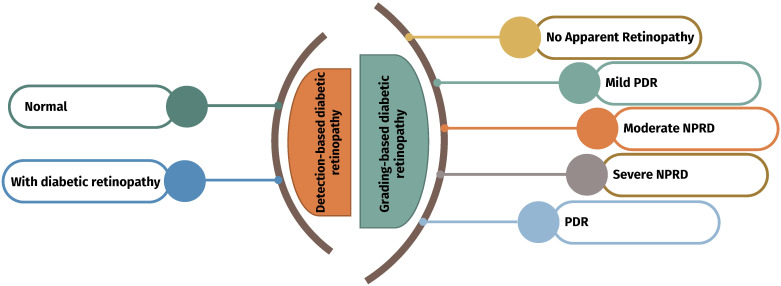
Types of diabetic retinopathy studies.

The diagnosis of diabetic retinopathy can be performed using two techniques: detection and grading. The detection of diabetic retinopathy is performed using binary classification (DR or normal retina), while diabetic retinopathy grading consists of detecting and annotating the infected parts, including the types of infection: mild, moderate, or severe. Figure 2 summarizes these two different types of DR studies. This section describes these studies by categorizing them into diabetic-retinopathy-detection-based studies and DR-grading-based studies. All these studies are summarized in Table 1.

### 2.1. DR-Detection-Based Studies

The diabetic retinopathy detection studies perform binary classification of the input images as healthy or DR. Here, we focus on deep-learning-based methods, which are the most effective approaches compared with other machine-learning-based or traditional techniques. For example, Kazakh-British et al. [10] proposed a simple convolutional neural network (CNN) to automatically classify DR. They used the original images and images filtered using an anisotropic diffusion filter in the experiments. From the obtained results, the authors found that the use of the anisotropic diffusion filter improved the performance. In the same context, the authors in [11,12,13,14] used CNN architectures to perform binary classification to identify the presence of diabetic retinopathy. After applying the Wiener filter to the fundus images and using OTSU for the segmentation, the authors of [15] proposed a deep CNN for multi-class classification of the fundus images into those having several vision-threatening diseases such as DR and the normal fundus images.

**Figure 3:**
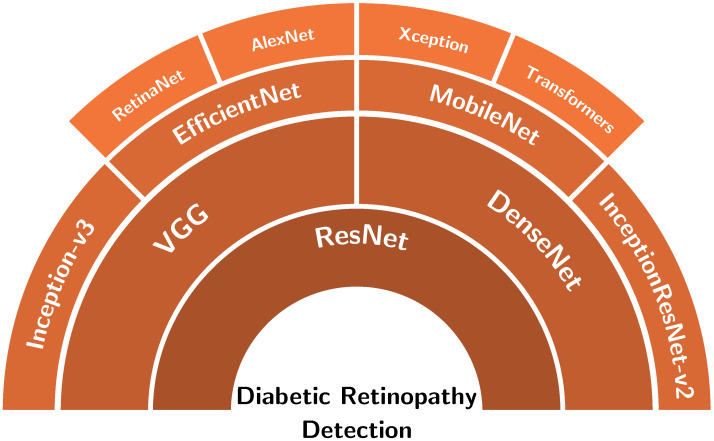
Backbones used for diabetic retinopathy detection studies.

Instead of using simple convolutional neural networks, some authors have used pre-trained models (backbones) for transfer learning or for feature extraction to implement their methods. These are shown in Figure 3. For example, InceptionV3 was used by the authors in [16] to classify DR on RGB and textures features. Umapathy et al. [17] used a pre-trained InceptionV3 to perform DR classification. A binary CNN (BCNN) was proposed by the authors in [18] for DR classification to reduce memory consumption and improve runtime. Both binomial classification and multinomial classification of fundus images were performed by the authors in [19] using the MobileNetV2 architecture since this architecture requires less training time and can be used in mobile systems. Saranya et al. [20] used the DenseNet-121 model to detect DR from fundus images, while transfer learning using EfficientNet-B0, EfficientNet-B4, and EfficientNet-B7 were exploited to detect DR in [21]. The same backbones were used in [22] to classify DR into referable/vision-threatening DR. The EfficientNet-B3 backbone initialized with ImageNet weights and fully connected layers initialized with HE initialization were used for training by the author in [23]. From the experiments, the EfficientNet model gave good results compared to the ground-truth.

Another Backbone was used by Sudarmadji et al. [24] for diabetic retinopathy detection. The proposed method used the VGG network for feature extraction to implement the proposed CNN-based model. Boral and Thorat [25] used a transfer learning approach using InceptionV3 followed by SVM to perform DR classification. In another paper, five transfer learning models, Xception, InceptionResNetV2, MobileNetV2, DenseNet-121, and NASNetMobile, were used by the authors in [26] to perform binary classification of DR. DenseNet-121 was used as the transfer-learning-based method by the authors in [27] to identify MAs, Exs, and hemorrhages from the input images to detect DR. Furthermore, transfer learning, VGG, AlexNet, Inception, GoogleNet, DenseNet, and ResNet were used by the authors in [28]. Another study [29] involved a comparison of three types of deep-learning-based architectures including Transformer-based networks, CNNs, and multi-layered perceptrons (MLPs) for DR classification. Different models included in the study were EfficientNet, ResNet, Swin-Transformer, Vision-Transformer (ViT), and MLP-Mixer. The models that are based on the transformer architecture were found to have the best accuracy among these. An ensemble model consisting of three CNN models was used by the authors in [30] for DR classification. It was based on stack generalization. ResNet-50 and VGG-16 were also used. Four vital features of using the CNN for DR classification, different architectures of the CNN, preprocessing techniques, class imbalance, and fine-tuning were evaluated by the authors in [31]. AlexNet, ResNet-50, and VGG-16 were employed for this purpose. The performances of twenty-eight deep hybrid architectures for binary classification of DR into referable DR and non-referable DR were empirically evaluated by the authors of [32]. This was compared with end-to-end deep learning (DL) architectures. A hybrid architecture using the SVM classifier and MobileNetV2 for feature extraction was found to be the best-performing among these. A three-class classification of fundus images into normal, glaucomatous, and diabetic retinopathy eyes was performed by the authors in [33]. Multiple CNN models—MobileNetV2, DenseNet-121, InceptionV3, InceptionResNetV2, ResNet-50, and VGG-16—were used for DR classification.

A model based on ResNet with gradient-weighted class activation mapping (Grad-CAM) was used by the authors of [34] for lesion detection and DR classification. The lesions included MAs, HEs, hemorrhages, and CWSs. Quellec et al. [35] found that, when training for image-level classification was used with ConvNet, it became capable of performing lesion detection. The training was performed with a simplification of the back-propagation method. The images were classified into non-referable DR and referable DR. A new neural network called the lesion-guided network (LGN) was proposed by Tang et al. [36] to diagnose DR. For lesion detection, the backbone was RetinaNet with ResNet-50. A lesion-aware module (LAM) was also used to improve the rough lesion maps. Enhanced DR detection was performed by using the Harris hawks optimization (HHO) algorithm along with a DCNN by the authors of [37]. Gunasekaran et al. [38] used a deep RNN (DRNN) to perform early detection of DR. A CNN-based method was proposed in [39] to detect DR. A very recent work [40] used seven different CNNs for DR diagnosis. Experiments in this study included single-modality and joint fusion strategies.

### 2.2. DR-Grading-Based Studies

**Figure 4:**
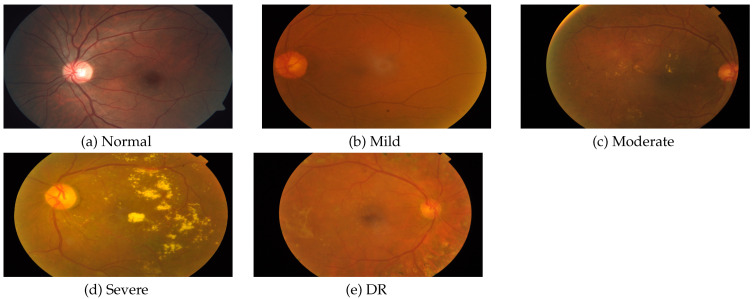
The five types of diabetic retinopathy.

As per the International Clinical Diabetic Retinopathy (ICDR) [41] scale, diabetic retinopathy can be graded into separate grades: no apparent retinopathy, mild non-proliferative diabetic retinopathy (NPDR), moderate NPDR, severe NPDR, and proliferative diabetic retinopathy (PDR). An example of each grade is presented in Figure 4. Many studies have been proposed for multi-class classification and grading of fundus images into the above-mentioned five stages.

A simple CNN model was used by the authors in [42] after applying a green channel filter to assess the stage of DR from fundus images. A CNN, which combined multi-view fundus images, was used along with attention mechanisms by the authors in [43]. It was called MVDRNet and used VGG-16 as the basic network. A locally collected dataset containing multi-view fundus images was employed for this. Another study that used a locally collected dataset from the University Hospital Saint Joan, Tarragona, Spain, is [44]. The CNN model used had batch normalization followed by the ReLU function. This was followed by a linear classifier and a softmax function. Two datasets—a balanced dataset with no augmentation and another one with augmentation—were used by the authors in [45]. A CNN was used to demonstrate the improvement in accuracy in DR grading due to the augmentation. Agustin and Sunyoto [46] performed a comparison of different regularization methods regarding how they reduce the overfitting of CNNs when used for DR severity grading. Dropout regularization was found to reduce overfitting and to increase accuracy.

**Table 1:** Retinopathy-grading-based studies during the period 2017–2020.

Method	Year	Method	Dataset(s)
Li et al. [47]	2017	CNN-based transfer learning, SVM	DR1 and MESSIDOR
Ardiyanto et al. [48]	2017	Deep-DR-Net	FINDeRS
Kwasigroch et al. [49]	2018	Transfer learning and VGG	Kaggle EyePACS
Wang et al. [50]	2018	AlexNet, VGG-16, and InceptionV3	Kaggle EyePACS
Zhou et al. [51]	2018	Inception-ResNet-v2, BaseNet	Kaggle EyePACS
Shrivastava and Joshi [52]	2018	InceptionV3, SVM	Kaggle EyePACS
Arora and Pandey [53]	2019	AlexNet, VGG-16, and InceptionV3	Kaggle EyePACS
Kassani et al. [54]	2019	InceptionV3, MobileNet, and ResNet-50	Kaggle APTOS
Hathwar and Srinivasa [55]	2019	Inception-ResNet-V2, and Xception	Kaggle EyePACS, IDRiD
Bellemo et al. [56]	2019	Ensemble of Adapted VGG and ResNet	Kitwe Central Hospital, Zambia
Kumar [57]	2019	Ensemble of GoogleNet, AlexNet, and ResNet-50	Kaggle EyePACS
Thota and Reddy [58]	2020	Pre-trained VGG-16	Kaggle EyePACS
Nguyen et al. [59]	2020	VGG-16 and VGG-19	Kaggle EyePACS
Lavanya et al. [60]	2020	ImageNet	Kaggle DR
Elzennary et al. [61]	2020	DenseNet-121	Kaggle APTOS
Barhate et al. [62]	2020	Autoencoder and VGG	Kaggle EyePACs
Wang et al. [63]	2020	Multichannel-based semisupervised GAN	MESSIDOR
Khaled et al. [64]	2020	VGG-16	Kaggle EyePACS
Islam et al. [65]	2020	Transfer Learning and VGG-16	Kaggle APTOS
Wang et al. [66]	2020	Hierarchical multi-task deep learning framework	Shenzhen, Guangdong, China
AbdelMaksoud et al. [67]	2020	E-DenseNet	Kaggle EyePACS, Kaggle APTOS
Yaqoob et al. [68]	2020	ResNet-50	MESSIDOR-2 and Kaggle EyePACS
Taufiqurrahman et al. [69]	2020	MobileNetV2, SVM	Kaggle APTOS
Vaishnavi et al. [70]	2020	AlexNet	Kaggle EyePACS
Shankar et al. [71]	2020	Synergic deep Learning (SDL) model	MESSIDOR
Karki and Kulkarni [72]	2021	EfficientNet	Kaggle APTOS
Qian et al. [73]	2021	Res2Net and DenseNet	Kaggle EyePACS
Shorfuzzaman et al. [74]	2021	CNN	Kaggle APTOS, MESSIDOR, IDRiD
Sugeno et al. [75]	2021	EfficientNet-B3	Kaggle APTOS, DIARETDB1
Lee and Ke [76]	2021	VGG-16 and ResNet-50	IDRiD
Nazir et al. [77]	2021	DenseNet-100, CenterNet	Kaggle APTOS, IDRiD
Xiao et al. [78]	2021	SE-MIDNet	Kaggle EyePACS
Li et al. [79]	2021	SAGN, GCNN	Kaggle APTOS, Kaggle EyePACS
Martinez-Murcia et al. [80]	2021	ResNet-18 and ResNet-50	MESSIDOR
Rajkumar et al. [81]	2021	ResNet-50	Kaggle EyePACS
Swedhaasri et al. [82]	2021	SE-ResNet-50, EfficientNet	Kaggle APTOS
Reguant et al. [83]	2021	InceptionV3, ResNet50, and Xception	Kaggle EyePACS, DIARETDB1
Hari et al. [84]	2021	Xception, InceptionV3, and DenseNet-169	Kaggle EyePACS
Saeed et al. [85]	2021	VGG-19, ResNet, and DPN107	Kaggle EyePACS, MESSIDOR
Jabbar et al. [86]	2022	VGG	Kaggle EyePACS
Shaik and Cherukuri [87]	2022	HA-Net	Kaggle APTOS, IDRiD
Chandrasekaran and Loganathan [88]	2022	ResNet and AlexNet	Kaggle EyePACS
Oulhadj et al. [89]	2022	DenseNet, InceptionV3, and ResNet-50	Kaggle APTOS
Nair et al. [90]	2022	VGG-16, ResNet-50, and EfficientNet-B5	Kaggle APTOS
Deepa et al. [91]	2022	Xception, InceptionV3, and ResNet-50	Kaggle DR, DIARETDB, STARE
Farag et al. [92]	2022	DenseNet-169 with CBAM	Kaggle APTOS
Canayaz [93]	2022	EfficientNet-B0, DenseNet-121	Kaggle APTOS
Bilal et al. [94]	2022	U-Net	Kaggle EyePACS, MESSIDOR-2
Murugappan et al. [95]	2022	DRNet	Kaggle APTOS
Chen and Chang [96]	2022	InceptionV3 and EfficientNet	Kaggle APTOS
Butt et al. [97]	2022	ResNet-18 and GoogleNet	Kaggle APTOS
Elwin et al. [98]	2022	DCNN, ShCNN	IDRiD, DDR
Deepa et al. [99]	2022	DCNN	Kaggle EyePACS, DIARETDB, STARE

A deep CNN model called DR|GRADUATE was presented by the authors in [100]. It was a new DL approach for DR grading, which could give a pathologically explainable description to support its judgment. It also provided an assessment of the ambiguity of its prediction. Feature extraction using a multipath CNN was used by the authors in [5]. After this, DR was graded using three different ML classifiers, SVM, random forest, and J48. Sugeno et al. [75] used the EfficientNet model to grade DR after using morphological operations and image processing for lesion detection. A multi-task model with EfficientNet-B5 was used by the authors of [101] for DR grading. Feature extraction performed with the EfficientNet backbone was fed to the dropout layer, which was followed by an ordinal regression section and a classification section. Shankar et al. [71] proposed a deep CNN model called the synergic deep learning (SDL) model to grade DR. Histogram-based segmentation was performed before this.

A pre-trained VGG-16 was used by the authors of [58] to train their proposed CNN to improve the accuracy of DR grading. VGG-16 and VGG-19 were used by the authors of [59] to grade DR. They mirrored and rotated the images to augment the dataset. The VGG-16 and ResNet-50 models were modified and used by the authors in [76] to grade DR with the help of the dropout concept. A cascaded model consisting of two VGG-16 models was used by the authors of [64]. The first model outputs “yes” or “no” to detect DR, and the second model classifies the fundus images into four different DR stages. Shaik and Cherukuri [87] used a model named “Hinge Attention Network (HA-Net)” which has multiple attention stages for DR severity grading. Initial spatial representations from the input images were extracted using a pre-trained VGG-16 base.

An automated DR detection system using a Raspberry Pi was developed by the authors of [60]. They used ImageNet for DR grading. Elzennary et al. [61] used the DenseNet-121 neural network architecture with the aid of transfer learning to determine the severity of DR. Both of these studies used the Python framework called Flask to create interfaces that can be used by doctors to detect DR. A custom CenterNet with DenseNet-100 support was used by the authors of [77] to detect eye diseases from retinal images. This study graded the severity of DR by separating the fundus images according to the lesions present.

Another classification network for DR-SE-MIDNet was introduced by the authors of [78]. It was built using an enhanced Inception module along with the squeeze-and-excitation (SE) module for grading. With the SE module, global information for the feature map on each channel was found. Feature extraction using InceptionV3 was performed using a hierarchical approach by the authors in [52]. The first layer was for binary classification into DR/no DR. The next one was to grade DR into the five DR stages. SVM with the radial basis function (RBF) kernel was utilized for classification. Wang et al. [63] used a multichannel-based semi-supervised GAN (SSGAN) for DR grading, which was capable of using labeled and unlabeled data as the training data. They used feature extraction to reduce the noise of the input images and for extracting the features of lesions. They also graded the lesions into three levels.

A new DL algorithm called Deep-DR-Net capable of being fit onto a small embedded board was introduced by the authors of [48] to grade DR. For this, they arranged a cascaded encoder–classifier network with a residual style to ensure that it was small in size. Li et al. [79] proposed a semi-supervised auto-encoder graph network (SAGN) to diagnose DR. In this, an autoencoder was used for feature learning. After this, the RBF was used to calculate neighbor correlations. Finally, a graph CNN (GCNN) was used to grade DR. A graph neural network (GNN), which extracts lesion ROI sub-images to emphasize only lesions in fundus images, was proposed by Sakaguchi et al. [102]. A graph is constructed from these sub-images for DR classification.

Transfer learning and the VGG architecture were used by Kwasigroch et al. [49]. For this reason, the ImageNet dataset was used to pre-train the VGG architecture. Another DL model that used transfer learning—VGG-16—was used along with a new color version preprocessing method by Islam et al. [65] for DR grading. ResNet-18 and ResNet-50 were used along with residual transfer learning by Martinez-Murcia et al. [80] for the same. Another transfer learning approach—the ResNet-50 architecture trained on the ImageNet dataset—was used for DR classification and grading by the authors in [81]. Another study that used transfer learning by fine-tuning using a well-annotated ImageNet dataset to train Inception-ResNet-V2 and Xception models was given in [55]. The latter was found to have better performance. CNN-based transfer learning followed by SVM were used by the authors of [47]. AlexNet and VGG were pre-trained using the ImageNet dataset. Features extracted with the help of transfer learning were provided to SVM for DR grading. An ensemble model consisting of SE-ResNeXt50, EfficientNet-B4, and EfficientNet-B5 along with transfer learning was used by the authors of [82] for DR grading. The InceptionV3, ResNet-50, InceptionResNet50, and Xception models were used for DR grading by the authors in [83]. The parameters were initialized using transfer learning. They created visualization maps to investigate the clinical significance of the decisions made by the CNN models. Wang et al. [50] used AlexNet, VGG-16, and InceptionV3 along with transfer learning for DR grading. InceptionV3 was found to provide the best accuracy in their study. Jabbar et al. [86] used a transfer-learning-based VGG architecture for DR grading. Various data augmentation techniques were used to balance the classes in the training data.

Experiments using several deep neural networks (DNNs) were carried out to yield algorithms that grade DR conforming to the ICDR standards by the authors in [103]. The network was also trained to make several other binary classifications. Synchronized diagnosis of DR severity, DR features, and referable DR was conducted by the authors of [66]. A hierarchical multi-task DL framework with a skip connection was utilized for automatically merging the DR-related feature output with DR severity analysis. An ensemble of two CNN architectures—a modified VGG and RNN—was utilized for grading DR by the authors in [56]. Apart from the grading of DR as per the ICDR scale, the images were classified into referable DR/vision-threatening DR. Xception, InceptionV3, and DenseNet-169 were used by the authors of [84] for DR grading. They used the Kaggle DR dataset and created two versions of it: balanced and imbalanced. The Xception model, which was trained using the imbalanced version of the dataset, was found to have the best performance. VGG-19, ResNet-152, and DPN107 were used with two-stage transfer learning by the authors in [85] for grading DR. The initial layers of the pre-trained models were adjusted for the preceding layers to understand the lesions and also the normal areas. Zhou et al. [51] used a multi-cell architecture, which could increase the depth of the DNN, as well as the resolution of the input image. A three-layer architecture that used Inception-ResNet-v2 and BaseNet to grade DR was proposed. AlexNet, VGG-16, and InceptionV3 were used by the authors in [53] to determine DR stage classification. Image augmentation techniques were used before training. The DR grading performance of three models, a shallow CNN, ResNet with soft attention, and AlexNet for DR using a new hyper-analytic wavelet (HW) phase activation function, was compared by the authors in [88]. AlexNet for DR was found to show the maximum improvement in performance in their experiments. Oulhadj et al. [89] applied a deformable registration to the retina and graded DR using four CNN models, DenseNet-121, Xception, InceptionV3, and ResNet-50. Three pre-trained models, VGG-16, ResNet-50, and EfficientNet-B5, were used for DR grading by the authors in [90]. ResNet-50 was found to perform best among the three. The performance of three pre-trained models, Xception, InceptionV3, and ResNet-50, in DR grading was compared by the authors of [91]. Their simulation result found the Xception model to perform better.

ResNet was used by the authors in [104] for feature extraction. After this, they used SVM, as well as a neural network (NN) pixelwise classifier to grade DR. AD2Net—a new CNN model having the qualities of Res2Net and DenseNet—was used by the authors of [73] for DR grading. An attention mechanism was used to make the network concentrate on understanding useful information from the images. A deep supervision of inception-residual network (DSIRNet) was used by [105], which was based on the network design ideas of GoogleNet and ResNet for feature extraction to grade DR. They also used a deep monitoring method to enhance the thermal classification effect of the training network. Yaqoob et al. [68] trained an optimized ResNet-50 having features from a canny edge detector and histogram of gradients to perform the grading of DR using two public datasets. An ensemble made of GoogleNet, AlexNet, and ResNet-50 was utilized by the authors of [57]. The images were preprocessed and fed to this ensemble model for DR grading. A CNN-based DL ensemble framework in which weights from distinct models were merged to make a solo model, which can extract prominent features from many lesions in the input images, was used by Shorfuzzaman et al. [74] to grade DR. Some CNN models that were pre-trained with the ImageNet dataset—the ResNet-50, DenseNet-121, Xception, and Inception models—were used for this. After preprocessing with CLAHE for segmentation, Vaishnavi et al. [70] used the AlexNet architecture for feature extraction. Finally, a softmax layer was utilized to grade the images according to DR severity.

An ensemble of five models from the EfficientNet family was used for DR grading by the authors in [72] by pre-training on ImageNet. These models were also used independently for the same, and EfficientNet-B3 performed better than the ensemble model and the other four models. A hybrid and effective model, MobileNetV2-SVM, was used by the authors of [69] to grade DR images. A stack of residual bottleneck layers, which consisted of a stack of bottleneck residual blocks, was used to construct the MobileNetV2 model. Jiang et al. [106] used three models—InceptionV3, ResNet-152, and Inception—ResNet-V2 to grade DR. An ensemble model consisting of these models, using the Adaboost algorithm, was also used. Another study used an embedded model consisting of five deep CNNs—ResNet-50, Xception, InceptionV3, DenseNet-121, and DenseNet-169 [107]. Stacked individual channels of the image were taken as the input. The forecast from separate models was averaged and used to fix the final target label. The green channel was found to give the best performance in grading DR. A novel hybrid DL model known as E-DenseNet was proposed by the authors of [67] to grade DR. It was a hybrid between a customized EyeNet and DenseNet based on DenseNet-121. The Xception deep feature extractor was used by the authors of [54] to advance the capability of the typical Xception architecture in classifying DR. They also used transfer learning along with hyper-parameter tuning.

A novel CNN model based on the DenseNet-169 architecture combined with a convolutional block attention module (CBAM) was used by the authors of [92] for DR severity classification. The ResNet-101 model was used for DR grading and to analyze the risk of macular edema by the authors in [108], and it was found to perform better than the ResNet-50 model. A heuristically constructed deep neural network was used by the authors of [109] to determine the severity levels of DR. An architecture consisting of an autoencoder along with a VGG network was used by the authors of [62] to reduce overfitting during DR detection. The network was pre-trained in a self-supervised manner.

The binary bat algorithm (BBA), equilibrium optimizer (EO), gravity search algorithm (GSA), and gray wolf optimizer (GWO) were used as the wrapper methods to select the best features that were obtained from the EfficientNet-B0 and DenseNet-121 models for DR grading by the authors in [93]. Transfer-learning-based InceptionV3 was used by the authors of [94] for DR grading. They used two separate U-Net models for OD and blood vessel segmentation. Five DL models—DenseNet-121, InceptionV3, ResNet-153, VGG-16, MobileNet, and InceptionResNet—were used with transfer learning for DR grading by the authors of [110]. Out of these, the VGG-16 model was found to provide the highest accuracy in their experiments. Deepa et al. [99] used a pre-trained Xception model along with hierarchical clustering of image patches by the Siamese network to grade DR fundus images. A boosting-based ensemble learning method followed by a CNN was used by the authors of [111] for DR grading. A novel few-shot classification framework called DRNet was used by the authors of [95] for DR detection and grading. Episodic training was used to train the model on few-shot classification tasks. Both DR detection and DR grading were performed by the authors of [112] using a Bayesian neural network (BNN). Experiments using nine BNNs were performed to utilize their capability of uncertainty estimation in classifying DR. Chen and Chang [96] used the InceptionV3 and EfficientNet models to grade fundus images according to DR severity. A novel hybrid model called E-DenseNet was used by the authors of [113] for DR grading. It was a combination of the EyeNet and DenseNet models based on transfer learning. Another study by the authors of [97] used a similar hybrid model based on transfer learning for the detection and grading of DR. The model consisted of ResNet-18 and GoogleNet. Ar-HGSO, which is an autoregressive-Henry gas-sailfish-optimization-enabled deep learning model was used by the authors of [98]. The DCNN was used for DR detection, and the Shepard CNN (ShCNN) was used for severity classification. Rajavel et al. [114] introduced a cloud-enabled DR grading system that used an optimized deep belief network (O-DBN) classifier model. Dimensionality reduction and noise removal were performed by them using the stochastic neighbor embedding (SNE) feature extraction approach. LeNet-5 was used by the authors in [115] for DR grading. A spiking neural network (SNN) was used for DR grading by the authors in [116]. They used the chimp optimization algorithm with DenseNet (COA-DN) for feature extraction.

Table 1 summarize the studies that were presented in this section.

## 3. Preprocessing Techniques Used to Grade DR Fundus Images

Image enhancement is performed in most DR studies with the help of several preprocessing techniques. Preprocessing can consist of several steps such as image variation attenuation, intensity conversion, denoising, and contrast enhancement [117]. The attenuation of fundus images is required since there will be a wide variation in the color of the retina of different patients. Intensity conversion is used to make the features clearly visible in an image. Denoising of fundus images is required since much noise may be introduced into these images during the image acquisition process. Finally, contrast enhancement is essential since retinal images captured with the help of a fundus camera will have maximum contrast at the image center, which gradually reduces when moving away from the center. Other common preprocessing steps include image resizing and performing several image augmentations using techniques such as rotation, flipping, and zooming.

## 4. DR Datasets

The success of all these DL studies relies greatly upon the datasets that are used. The quality of the dataset used and the precision of the annotations will have a huge impact on the results that will be obtained by these methods. Hence, we created a list of some commonly used fundus image datasets for DR diagnosis. Table 2 presents this list.

A few of the commonly used publicly available datasets in these studies are STARE, IDRiD, MESSIDOR, DIARET DB1, the Kaggle APTOS dataset, and the Kaggle EyePACS dataset. Out of these, Kaggle’s EyePACS and APTOS datasets are the most widely used datasets for DR detection/grading. However, these contain fundus images taken with different cameras and settings. The largest among these is the Kaggle EyePACS dataset with more than 88,000 fundus images, whereas some datasets, such as DIARETDB1, HRF, and DRiDB, have less than 100 fundus images.

Almost all of them are annotated for DR detection, while some datasets such as MESSIDOR and Kaggle EyePACS have been annotated also for DR grading. Most of the studies used different datasets/combinations of datasets for training and validation purposes since most of the datasets are small in size. However, some studies have used their own locally collected datasets for their experiments [43,44].

## 5. Discussion

In order to evaluate the diabetic retinopathy detection and grading methods on different datasets, a set of metrics is used, including model accuracy, sensitivity, sensitivity, and the AUC. These metrics are generally the most-used ones for detection and segmentation in computer vision tasks. In this section, we present the obtained results per dataset using the cited method for detection and grading methods. These results are reported in tables and figures in order to show the most-performed techniques using different architectures.

**Figure 5:**
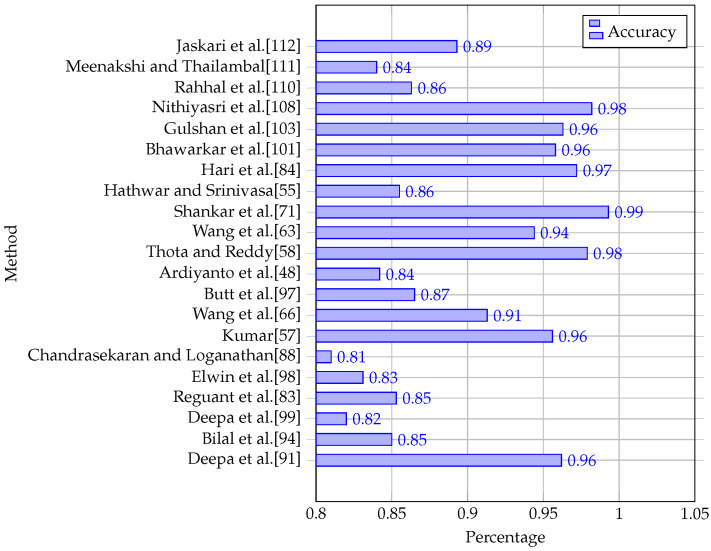
Performance comparison of diabetic-retinopathy-grading-based studies that used the Kaggle APTOS dataset.

**Figure 6:**
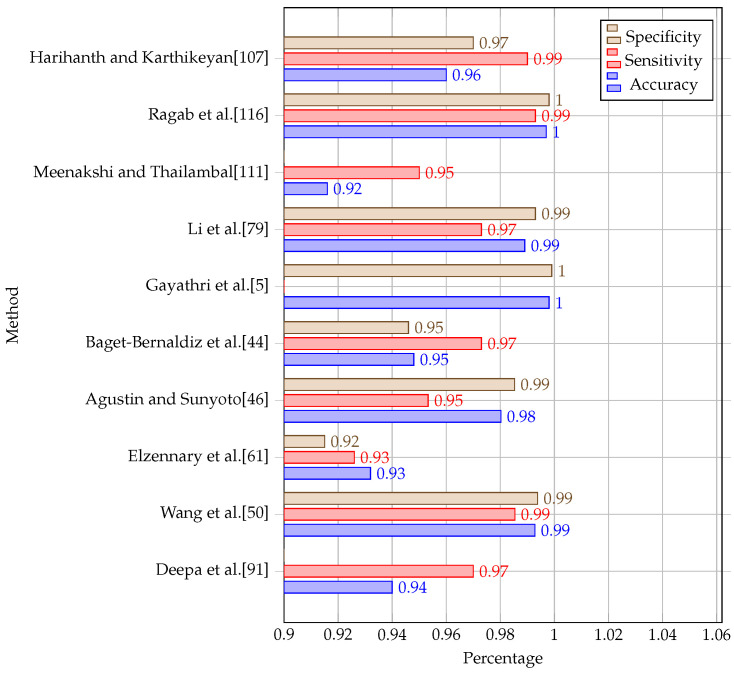
Performance comparison of diabetic-retinopathy-grading-based studies that used the MESSIDOR dataset.

Table 3 and Table 4 and Figure 5 and Figure 6 show a comparison of the results obtained by some of the studies that have been reviewed. Studies that have used the same publicly available datasets have been grouped for comparison. Kaggle APTOS and Kale EyePACS are the largest datasets that have enabled these researchers to perform their experiments.

### 5.1. Diabetic Retinopathy Detection

Diabetic retinopathy detection methods are performed on datasets of two classes that represent the images with diabetic retinopathy and the images without diabetic retinopathy. To show that, Table 3 compares some DR-detection-based studies. The most studies used Kaggle’s APTOS and EyePACS datasets, due to their size, which is large compared to the others. The binary classification to detect the fundus images that have DR lesions and, thus, detect the presence of DR is performed by the proposed methods. For that reason, we can see that all the methods can classify diabetic retinopathy with good performance in accuracy, while the sensitivity and specificity values were not mentioned in some of the studies. From the table of the obtained results using the proposed method on the Kaggle APTOS dataset, we can find that the authors in [11] achieved the best accuracy value of 94% with a difference of 4% better than the accuracy obtained using [40] and more than 8% for the other methods. Using the sensitivity and specificity metrics, the method in [22] achieved the best results. On the MESSIDOR and MESIDOR2 datasets, the methods used in [21,24] achieved the best accuracy, respectively. However, we can see that, for MESSIDOR2, the accuracies were lower than the obtained accuracies on MESSIDOR, due to the fact that the size of MESSDOR2 is larger than MESSIDOR, which can explain the difference between the accuracy on MESSDOR2 being 91% and 99% on MESSIDOR. The same observation is made for Kaggle EyePACS, which is a large-scale dataset; the accuracy performances were generally less than 91%, except for [18,24,25], which achieved an accuracy of up to 97%. For all the datasets including STARE, HRF, and IDRid, the performance of the proposed methods needs improvements due to the importance of the topic, as well as the impact of the error if these techniques are used in real-world diagnostics.

### 5.2. Diabetic Retinopathy Grading

Diabetic-retinopathy-grading-based studies comprise another classification category for diabetic retinopathy analysis. The proposed methods for diabetic retinopathy grading are based on deep learning using different CNN architectures. For that, transfer learning has been widely used in the reviewed studies. This is due to the effectiveness of the known backbones for the image classification tasks. This includes deep learning architectures/models such as encoder–decoder, VGG, DenseNet, Inception, Xception, EfficientNet, graph neural networks, etc. In addition, preprocessing techniques were also used in different studies to improve performance, as mentioned in Section 3. Grayscale conversion, resizing, CLAHE, and green channel extraction are some commonly preferred preprocessing techniques.

These techniques aid in improving the feature extraction process by removing unnecessary noise from the images.

In this section, we attempt to present the grading-based methods on popular DR datasets. The evaluation used a set of metrics including the accuracy, sensitivity, and specificity. Table 4 presents a comparison of the obtained results using the proposed method on studies that have used the Kaggle EyePACS, MESSIDOR2, DDR, and IDRid datasets. Figure 5 and Figure 6 illustrate the experimental results using the proposed methods on the Kaggle APTOS and MESSIDOR datasets. From Table 4, we can find that the proposed methods succeeded in achieving high accuracies on MESSIDOR2, DDR, IDRid, reaching up to 97%. The same observation is made for the other metrics including the sensitivity and specificity. On Kaggle EyePACS, the proposed method in [5] achieved the best accuracy, as well as the best specificity metric value, while we can find that the majority of the methods achieved an accuracy of less than 90%. This is due to the complexity and size of the dataset. On Kaggle APTOS, from the obtained results represented in Figure 5, we can find that most methods that used accuracy as an evaluation metric achieved an accuracy of less than 97%, while only the method in [71] achieved an accuracy of 99%. For the MESSIDOR dataset, the proposed methods used the accuracy, sensitivity, and specificity metrics to evaluate their results. The obtained results are presented in Figure 6. It shows that many methods achieved an accuracy of up to 99% including [5,50,79,116], while the others achieved an accuracy of up to 92%.

From the presented results on different datasets, we can conclude that some of the methods such as [5] succeeded in classifying diabetic retinopathy with grading-based and detection-based methods with high accuracies, while some of the proposed methods were good for some datasets and less efficient for others. This makes diabetic retinopathy classification a challenging task even with the improvements achieved during the last ten years using different deep learning techniques.

**Table 4:** Performance comparison of diabetic-retinopathy-grading-based studies that used the Kaggle APTOS, MESSIDOR2, DDR, and IDRid datasets. The **bold** and underlined fonts, respectively, represent **first** and second place.

Dataset	Method	Accuracy	Sensitivity	Specificity
MESSIDOR 2	Yaqoob et al. [68]	**0.970**	-	-
	Bilal et al. [94]	0.946	**0.948**	**0.944**
DDR	Rahhal et al. [110]	**1.00**	-	-
	Elwin et al. [98]	0.914	**0.925**	**0.905**
IDRid	Shorfuzzaman et al. [74]	0.923	**0.980**	-
	Elswah et al. [104]	0.866	-	-
	Sakaguchi et al. [102]	0.793	-	-
	Gayathri et al. [5]	**0.990**	-	**0.997**
	Lee and Ke [76]	0.972	0.702	0.921
	Nazir et al. [77]	0.981	-	-
	Shaik and Cherukuri [87]	0.664	-	-
	Nithiyasri et al. [108]	0.977	0.978	0.989
	AbdelMaksoud et al. [113]	0.930	0.967	0.720
	Elwin et al. [98]	0.914	0.925	0.905
	Sri et al. [115]	0.970	-	-
Kaggle EyePACS	Vaishnavi et al. [70]	0.958	0.920	0.978
	Thota and Reddy [58]	0.740	0.800	0.650
	Barhate et al. [62]	0.762	-	-
	Kwasigroch et al. [49]	0.508	-	-
	Wang et al. [50]	0.632	-	-
	Zhou et al. [51]	0.632	-	-
	Shrivastava and Joshi [52]	0.818	-	-
	Arora and Pandey [53]	0.744	-	-
	Kumar [57]	0.699	-	-
	Maistry et al. [45]	0.870	-	-
	Nguyen et al. [59]	0.820	0.800	0.820
	Khaled et al. [64]	0.631	-	-
	Harihanth and Karthikeyan [107]	0.819	-	-
	AbdelMaksoud et al. [113]	0.968	0.983	0.72
	Yaqoob et al. [68]	0.979	-	-
	Qian et al. [73]	0.832	-	-
	Gayathri et al. [5]	**0.999**	-	**1.00**
	Xiao et al. [78]	0.882	**0.994**	0.976
	Li et al. [79]	0.944	0.840	0.822
	Rajkumar et al. [81]	0.894	0.987	0.999
	Reguant et al. [83]	0.950	0.860	0.960
	Hari et al. [84]	0.830	-	-
	Saeed et al. [85]	0.997	0.960	0.998
	Jabbar et al. [86]	0.966	-	-
	Chandrasekaran and Loganathan [88]	0.980	0.990	-
	Bilal et al. [94]	0.979	0.969	0.969
	Deepa et al. [99]	0.960	-	-

## 6. Future Directions

Finally, we would like to provide some future research directions that were identified during this study. The latest trends such as using interpretable AI and cloud-enabled systems are also being used by some researchers in this field, as well as in medical imaging analysis [118,119,120,121]. Since interpretation will be preferred by doctors to diagnose DR, more studies on explainable AI may come up in the future such as those by Shorfuzzaman et al. [74] and Chetoui and Akhloufi [22]. Such DR-diagnosing systems will be able to help doctors rely on them with more confidence. The use of cloud-enabled systems for computer-aided DR detection systems such as the one by Rajavel et al. [114] will improve scalability. This will enable the development of large-scale systems for DR diagnosis.

Furthermore, developing low-cost standalone DR detection systems such as the one developed by the authors in [60] using a Raspberry Pi will be useful for deployment at health centers at a lower cost. Similar low-cost systems can also be created by developing DR diagnosis systems using smartphone-based retinal imaging systems such as the one by the authors in [122].

Another possible research direction is to develop more automated systems that are capable of determining more than one condition of the eyes, for example systems capable of diagnosing DR, as well as other conditions of the eyes such as glaucoma and diabetic macular edema, such as the one by the authors in [123].

## 7. Conclusions

In this work, we reviewed recent deep-learning-based approaches for diabetic retinopathy detection/diagnosis performed on fundus images. We classified the studies in this field into two categories including DR-detection-based studies and DR-severity-grading-based studies. Most studies graded fundus images into the severity levels suggested by the ICDR.

Almost all of the latest DL networks have been used efficiently by different studies for DR detection and grading. It was also noticed that there has been a considerable increase in the number of studies in this field recently. A list of the commonly used retinal fundus image datasets for DR detection and grading was also created in this study. Similar studies from each of the two categories of DR studies were compared according to their performance using the commonly used metrics of accuracy, sensitivity, and specificity. In future work, we will make a similar survey about the latest DR segmentation and lesion detection studies that have used DL.

## Figures and Tables

**Table 2:** Diabetic retinopathy datasets.

Dataset	No. of Images	Image Size
STARE	400	700 × 605
IDRiD	516	4288 × 2848
MESSIDOR	1200	Different sizes
HRF	45	3504 × 2336
Kaggle EyePACS	88,702	Different sizes
Kaggle APTOS 2019	5590	Different sizes
MESSIDOR 2	1748	Different sizes
DDR	13,673	Different sizes

**Table 3:** Performance Comparison of diabetic-retinopathy-detection-based studies. The **bold** and underlined fonts, respectively, represent **first** and second place.

Dataset	Method	Accuracy	Sensitivity	Specificity
Kaggle APTOS	Anoop et al. [11]	**0.946**	0.860	0.960
	Pamadi et al. [19]	0.780	-	-
	Saranya et al. [20]	0.830	-	-
	Chetoui and Akhloufi [22]	-	**0.991**	**0.972**
	Sanjana et al. [26]	0.861	0.854	0.875
	Kumar and Karthikeyan [29]	0.864	-	-
	Lahmar and Idri [32]	0.890	-	-
	El-Ateif and Idri [40]	0.907	0.928	0.893
MESSIDOR	Rego et al. [16]	-	0.808	0.973
	Umapathy et al. [17]	0.944	-	-
	Sudarmadji et al. [24]	**0.997**	**0.990**	**0.980**
	Hossen et al. [27]	0.949	0.926	0.971
	Qomariah et al. [28]	0.958	-	-
MESSIDOR2	Mudaser et al. [21]	**0.910**	-	-
	Sanjana et al. [26]	0.861	**0.854**	0.875
	Lahmar and Idri [32]	0.841	-	-
	El-Ateif and Idri [40]	0.777	0.310	**0.938**
Kaggle EyePACS	Saranya et al. [20]	0.830	-	-
	Jiang et al. [14]	0.757	-	-
	Kaushik et al. [30]	0.979	-	-
	Boral and Thorat [25]	**0.988**	0.977	**1.00**
	Rego et al. [16]	-	0.808	0.973
	Kolla and Venugopal [18]	0.910	-	-
	Chetoui and Akhloufi [22]	-	**0.981**	0.989
	Sudarmadji et al. [24]	0.984	0.980	0.970
	Lian et al. [31]	0.790	-	-
	Lahmar and Idri [32]	0.840	-	-
	Quellec et al. [35]	0.954	-	-
STARE	Kazakh-British et al. [10]	0.600	-	-
	Umapathy et al. [17]	**0.944**	-	-
HRF	Chakrabarty [13]	**1.00**	**1.00**	-
	Umapathy et al. [17]	0.944	-	-
IDRid	Nasir et al. [12]	**0.960**	**0.829**	-

## Data Availability

Not applicable.

## Sample Availability

Samples of the compounds...are available from the authors.

## Abbreviations

The following abbreviations are used in this manuscript:

DR	Diabetic retinopathy
DL	Deep learning
AI	Artificial intelligence
CNN	Convolutional neural network
